# Editorial: Synthetic biology and metabolomics: novel insight in oncology research

**DOI:** 10.3389/fbioe.2025.1768397

**Published:** 2026-01-06

**Authors:** Ruby Dhar, Karthikeyan Pethusamy, Manash K. Paul, Subhradip Karmakar

**Affiliations:** 1 Department of Biochemistry, All India Institute of Medical Sciences, New Delhi, India; 2 Department of Biochemistry, Manipal Academy of Higher Education, Manipal, India; 3 Department of Radiation Biology and Toxicology, Manipal School of Life Sciences, Manipal Academy of Higher Education, Manipal, India

**Keywords:** choline metabolism pathways, immunity, inflammation, microenvironmental dynamics, oncogenesis, tumor heterogeneity

## Metabolic reprogramming as a central driver of cancer progression

The comprehensive pan-cancer analysis by Zhang et al. reveals UBD/FAT10 as a critical nexus linking inflammation, immunity, and oncogenesis across 33 cancer types.

The comprehensive pan-cancer analysis by Zhang et al. reveals UBD/FAT10 as a critical nexus linking inflammation, immunity, and oncogenesis across 33 cancer types. Ubiquitin D (UBD/FAT10) emerges as a pivotal yet paradoxical player in cancer biology, exhibiting context-dependent roles across malignancies. Their integrative multi-omics investigation across 33 cancer types reveals UBD’s divergent expression patterns—upregulated in 14 cancers while downregulated in thyroid and kidney chromophobe carcinomas—challenging the notion of universal oncogenic drivers. The striking dichotomy in survival outcomes underscores UBD’s complexity, protective in melanoma and sarcoma, yet detrimental in uveal melanoma and pancreatic adenocarcinoma. This differential prognostic value suggests tissue-specific mechanisms that warrant deeper investigation. Notably, UBD’s association with IFN-γ-dominant immune landscapes and CD8^+^ T cell infiltration positions it at the immunometabolic crossroads of tumour-host interactions. Their experimental validation in esophageal carcinoma demonstrates UBD’s pro-tumorigenic effects via TP53 pathway modulation, offering mechanistic clarity beyond correlative observations. The identification of imatinib and TTNPB through molecular docking presents actionable therapeutic avenues for UBD-high tumors. This work exemplifies synthetic biology’s promise in cancer research, integrating computational predictions with experimental validation to decode molecular complexity. As precision oncology advances, understanding context-dependent biomarkers like UBD becomes essential for developing tailored therapeutic strategies that account for tumor heterogeneity and microenvironmental dynamics.

Complementing this work, Cao et al. elucidate how CHML drives hepatocellular carcinoma (HCC) progression through coordinated transcriptional and metabolic reprogramming. Their discovery of the CHML-SLC44A3-choline metabolic axis represents a significant advance in understanding HCC metabolism. Through integrated transcriptomics and untargeted metabolomics, they identified 591 differentially expressed genes and significant alterations in choline metabolism pathways. The demonstration that CHML upregulates SLC44A3 to enhance choline uptake, thereby increasing phosphatidic acid production and activating MAPK and PI3K-AKT signaling cascades, providing a mechanistic framework for therapeutic intervention. Their finding that CHML knockout significantly inhibits migration and invasion without affecting proliferation suggests a specific role in metastatic progression, offering potential for targeted anti-metastatic therapies in HCC.

The clinical translation of metabolic insights is elegantly demonstrated by Li et al., who employed LC-QTOF/MS-based metabolomics coupled with machine learning to differentiate benign, lymphoma, and metastatic superficial lymph nodes. Their analysis of 69 patients (lymph nodes divided into benign (N = 20), lymphoma (N = 29), and metastasis (N = 20) groups) revealed 174 metabolites with significant changes, achieving 87.4%–89.3% diagnostic accuracy using linear discriminant analysis, thereby significantly outperforming conventional ultrasonography (63.1%–75.4%). The identification of specific metabolic signatures is particularly noteworthy: lymphoma nodes showed elevated nucleotides (inosine monophosphate increasing >3-fold), while metastatic nodes exhibited higher carbohydrates (sucrose, gentiobiose, raffinose, melezitose) and acylcarnitines. The correlation analysis, revealing 366 significant associations between metabolites and ultrasound features, suggests that metabolic changes directly influence tissue architecture, providing a biological basis for imaging characteristics.

## From association to causation: unravelling complex metabolic relationships

Yang et al. provide crucial causal evidence through Mendelian randomization analysis using GWAS data from over 170,000 individuals. Their two-step MR approach demonstrates that body mass index (BMI) influences breast cancer risk through distinct pathways: increasing ER + breast cancer risk via bioavailable testosterone (mediation effect β1×β2 = 0.025) while decreasing overall breast cancer risk through 35 metabolite pathways. The identification of differential effects on various cholesterol forms (particularly HDL cholesterol) and triglyceride levels resolves longstanding paradoxes in epidemiological observations. Their finding that BMI shows negative causal relationships with breast cancer (β = −0.184, q = 3.32E-03), ER + breast cancer (β = −0.183, q = 4.28E-03), and ER-breast cancer (β = −0.349, q = 1.03E-03) challenges conventional assumptions and highlights the importance of considering both direct and mediated effects in complex metabolic relationships.

## The tumor microenvironment: beyond cellular targets

Zhou et al. provide a comprehensive analysis of 28 distinct collagen types in tumor biology, revealing their multifaceted roles beyond structural support. Their review demonstrates that COL1, comprising approximately 30% of total body protein, creates both physical and biochemical barriers to therapy. The dual nature of collagen’s effects is particularly evident: while COL1 promotes tumor growth through CD133-p85 interaction and Akt phosphorylation in glioblastoma stem cells, COL3 exhibits tumor-suppressive effects in triple-negative breast cancer, with higher COL3:COL1 ratios associated with improved survival. The emerging therapeutic strategies they discuss—including collagenase treatment, lysyl oxidase inhibition, and collagen-binding domain engineering—represent promising approaches for overcoming the extracellular matrix barriers that limit drug penetration and immune cell infiltration. Their analysis of collagen crosslinking as a determinant of tissue stiffness provides mechanistic insights into how the physical properties of the tumor microenvironment influence cancer progression and therapeutic resistance.

## Immune checkpoint inhibitors and autoimmune complications

Yu et al. present a systematic review and meta-analysis of 13 studies encompassing 2,059 patients, revealing that 66.7% (95% CI: 45.1%–85.5%) of patients with baseline positive thyroid antibodies develop thyroid immune-related adverse events (irAEs) following immune checkpoint inhibitor treatment. Their analysis demonstrates significant geographical variations, with Asian populations showing 55.1% incidence versus 90.5% in non-Asian regions. The trend toward higher risk with thyroglobulin antibody positivity compared to thyroid peroxidase antibody positivity (OR = 1.83, 95% CI: 0.87-3.85), though not statistically significant, suggests potential for risk stratification. These findings have immediate clinical implications for patient monitoring and management, particularly given the association between thyroid irAEs and improved survival outcomes in certain cancers.

## Navigating the governance challenges of converging technologies

Undheim comprehensive analysis of AI-enabled synthetic biology governance, based on 204 sources from 111 journals, presents a sobering assessment of regulatory preparedness. The proposed “whack-a-mole governance” framework captures the dynamic challenges across six governance levels (global, national, corporate, lab, scientist, citizens) and four governance types (precautionary, stewardship, bottom-up, laissez-faire). The identification of 1,297 unique keywords clustered into 81 categories illustrates the terminological confusion hampering effective communication and regulation. Particularly concerning is the finding that only 81 of 169 peer-reviewed papers explicitly discussed AI’s impact on synthetic biology, suggesting a disconnect between technological capabilities and risk assessment.

The literature reveals critical gaps in the Dual Use Research of Concern (DURC) regime, especially regarding generative AI’s potential to democratize access to advanced biological capabilities. Undheim notes that while the Biosafety in Microbiological and Biomedical Laboratories (BMBL) guidelines span 574 pages and the WHO’s Laboratory Biosafety Manual comprises 128 pages, neither adequately addresses AI-related biosafety concerns. With 64 BSL-4 labs globally and over 88,000 ILAC-accredited laboratories, the scale of potential risk amplification through AI-enabled capabilities is substantial.

## Integration of technologies and future directions

The convergence of these technologies creates synergistic opportunities beyond individual contributions. The success of machine learning in metabolomic diagnostics (Li et al.) combined with the identification of metabolic vulnerabilities in cancer (Zhang et al.), (Cao et al.) suggests that AI-driven metabolic profiling could accelerate drug discovery and personalized therapy selection. The O2PLS analysis by Cao et al. identifying top gene-metabolite associations (ADAP1, SLC1A1, LAMB3 among genes; limaprost, N-alpha-acetyl-l-lysine among metabolites) exemplifies how multi-omics integration can reveal novel therapeutic targets.

Several critical directions emerge from these collective works:

1. Precision Metabolic Targeting: The context-dependent effects of UBD across cancer types (Zhang et al.) and the specific metabolic dependencies revealed in HCC (Cao et al.) emphasize the need for tumor-specific and potentially patient-specific metabolic interventions. The development of metabolic signatures for different cancer subtypes could guide therapeutic selection.
2. Multi-modal Integration: The correlation between metabolites and ultrasound features (Li et al.) suggests opportunities for developing hybrid diagnostic approaches combining metabolomics with imaging. The 366 significant correlations identified between metabolic profiles and imaging characteristics could enable non-invasive metabolic assessment.
3. Causal Inference Frameworks: The successful application of Mendelian randomization (Yang et al.) to disentangle complex metabolic relationships provides a template for investigating other paradoxical associations in cancer epidemiology. This approach could be extended to understand the metabolic basis of therapeutic resistance and treatment outcomes.
4. Microenvironment Engineering: The comprehensive understanding of collagen biology (Zhou et al.) and its role in creating both physical and biochemical barriers suggests opportunities for combination therapies. Strategies that simultaneously degrade collagen barriers while delivering targeted therapies could significantly improve treatment efficacy.
5. Biomarker Development: The high diagnostic accuracy achieved through metabolomics (Li et al.) and the prognostic value of thyroid antibodies in ICI treatment (Yu et al.) highlight the potential for metabolic and immunological biomarkers in clinical decision-making.

## Challenges and opportunities in translation

Despite these advances, significant challenges remain in translating these discoveries into clinical practice. The heterogeneity observed across studies—from the variable effects of UBD in different cancers (Zhang et al.) to geographical variations in ICI-induced thyroid dysfunction (Yu et al.)—underscores the complexity of biological systems and the need for large-scale validation studies. The governance challenges identified by Undheim are particularly pressing given the rapid pace of technological advancement and the potential for dual-use applications. The economic considerations are substantial. As Undheim notes (Undheim), achieving pilot-scale biomanufacturing would require over $1 billion for a dozen facilities in the U.S. alone, with commercial production requiring fermentation capacity of 100,000L or more. The promise of a $4-30 trillion bioeconomy by decade’s end remains contingent on overcoming technical, regulatory, and scaling challenges.

## Conclusion and call to action

The articles in this Research Topic collectively demonstrate that we stand at an inflexion point in biomedical research and biomanufacturing. The convergence of AI, metabolomics, and synthetic biology offers unprecedented opportunities for understanding and treating cancer, developing novel therapeutics, and creating sustainable biomanufacturing processes. However, realizing this potential requires coordinated efforts across multiple fronts:

### Scientific

Continued development of integrated multi-omics approaches, standardization of data formats and analytical pipelines, and validation of findings across diverse populations.

### Clinical

Translation of metabolic insights into diagnostic tools and therapeutic strategies, development of biomarker-guided treatment algorithms, and implementation of precision medicine approaches.

### Regulatory

Development of adaptive governance frameworks that balance innovation with safety, updating of biosafety protocols to address AI-enabled capabilities, and international coordination on dual-use research oversight.

### Educational

Training of interdisciplinary scientists capable of working at the intersection of biology, chemistry, and computational sciences, and development of responsible research practices for emerging technologies.

As we navigate this transformative period, the need for continued dialogue between researchers, clinicians, policymakers, and the public becomes ever more critical. Only through such collaborative efforts can we ensure that these powerful technologies are developed and deployed in ways that maximize benefit while minimizing risks to human health and society.

